# Horses show individual level lateralisation when inspecting an unfamiliar and unexpected stimulus

**DOI:** 10.1371/journal.pone.0255688

**Published:** 2021-08-05

**Authors:** Paolo Baragli, Chiara Scopa, Martina Felici, Adam R. Reddon

**Affiliations:** 1 Department of Veterinary Sciences, University of Pisa, Pisa, Italy; 2 Research Center “E. Piaggio”, University of Pisa, Pisa, Italy; 3 Italian National Reference Centre for Animal Assisted Interventions, Istituto Zooprofilattico Sperimentale delle Venezie, Legnaro, Padua, Italy; 4 School of Biological and Environmental Sciences, Liverpool John Moores University, Liverpool, United Kingdom; University of New England, AUSTRALIA

## Abstract

Animals must attend to a diverse array of stimuli in their environments. The emotional valence and salience of a stimulus can affect how this information is processed in the brain. Many species preferentially attend to negatively valent stimuli using the sensory organs on the left side of their body and hence the right hemisphere of their brain. Here, we investigated the lateralisation of visual attention to the rapid appearance of a stimulus (an inflated balloon) designed to induce an avoidance reaction and a negatively valent emotional state in 77 Italian saddle horses. Horses’ eyes are laterally positioned on the head, and each eye projects primarily to the contralateral hemisphere, allowing eye use to be a proxy for preferential processing in one hemisphere of the brain. We predicted that horses would inspect the novel and unexpected stimulus with their left eye and hence right hemisphere. We found that horses primarily inspected the balloon with one eye, and most horses had a preferred eye to do so, however, we did not find a population level tendency for this to be the left or the right eye. The strength of this preference tended to decrease over time, with the horses using their non-preferred eye to inspect the balloon increasingly as the trial progressed. Our results confirm a lateralised eye use tendency when viewing negatively emotionally valent stimuli in horses, in agreement with previous findings. However, there was not any alignment of lateralisation at the group level in our sample, suggesting that the expression of lateralisation in horses depends on the sample population and testing context.

## Introduction

Animal brains exhibit anatomical and functional asymmetries [1, 2]. This feature is widespread among both vertebrate and invertebrate taxa suggesting adaptive significance [3]. The specialisation of the right and left hemisphere for different tasks is especially evident in the lateralisation of the sensory systems, including sight, audition, and olfaction [4–7]. The left hemisphere has been implicated in the analytic categorization of stimuli, while the right hemisphere, often takes precedence when responding to threats, during the detection of and escape from predators, and for emotional evaluation [8–10]. Thus, the right hemisphere seems to dominate in emotional attention [11], wherein a strong reaction is triggered by the stimulus which attracted the animal’s attention [12].

Historically, research on lateralisation has focused on humans [13] and non-human primates [14, 15], partly due to the mistaken belief that other animals did not show lateralised functioning of the brain [16]. Over the past three decades, studies on functional lateralisation have been extended to other species [17–20]. These studies have largely focused on bird [21] and fish models [22] because these animals have laterally placed eyes with monocular fields that project primarily to the contralateral hemisphere, allowing hemisphere use to be estimated by measuring eye use [23]. More recently, there has been a significant increase in studies on lateralisation in ungulates, in part because of the presence of similar anatomical advantages [24]. Among ungulates, the species that has undoubtedly been most closely examined is the horse (*Equus caballus*), likely owing to its recreational and social role in human society [24]. As with other ungulates, horses’ eyes are positioned on the sides of the head and horses see the world primarily through monocular vision. Their monocular visual field has a mean range of 190°-195° [25, 26] and therefore, horses must be able to process visual information, including monitoring for potential threats, from each monocular field. The visual system of the horse shows extensive decussation of the afferent sensory fibres to the contralateral hemisphere [27, 28]. Collectively, these characteristics make the horse a good model for investigating sensory lateralisation in mammals.

Threatening stimuli are primarily attended to with the left eye in many species of fishes, amphibians, reptiles, birds, and mammals [29, 30]. In accordance with this, horses show a tendency to use the left eye when examining unfamiliar objects [31]. Likewise, horses show overt escape reactions when a frightening stimulus is perceived with the left eye [32]. Attentional state, emotional reactivity, and competitive interactions are more intense when occurring on the left side [33]. This left side visual bias for negatively valent or anxiety inducing stimuli extends to applied management and husbandry contexts. Horses use their left eye to look at humans and objects during handling and riding procedures [34] and show higher emotional reactivity when approached from the left side by human handlers [35]. Human faces showing negative emotional expressions are preferentially observed by horses with the left eye [36].

In contrast to the aforementioned support for the predominance of the left eye and right hemisphere for processing negatively emotionally valent stimuli, there have been several reports of contradictory results [24] in a diversity of animal species [20, 37–39] including horses [4, 33, 34, 40–47]. These apparently incongruous findings may be attributable to variation in motivation or attention in the animals, which may affect the expression of sensory laterality [8, 48]. In horses, aspects of management, such as training and handling history may also influence the lateralisation of behaviour [31, 35].

Horses have a wide monocular field of vision and evaluating their current visual focus is not straightforward [26]. The scoring of eye use, regardless of whether in person or on video, maybe be susceptible to perspective errors due to the positioning of the camera or observer which could affect the attribution of the eye used to examine the stimulus. If the observer is not directly in line with the stimulus and the body axis of the animal the apparent eye use may be difficult to assign, potentially increasing variation [49–51]. Many published studies on horses do not provide a clear description of how such perspective errors were avoided.

Another variable which may affect the degree to which the right hemisphere appears to control the response to stressful stimuli is the nature of the stimulus used to induce a negative emotional state. Many studies assume a stress response is induced following the exposure to a noxious stimulus [52] or define a stimulus as aversive *a priori* and interpret the response as a stress reaction [53]. Each of these approaches could incorrectly classify a stimulus as a stressor and therefore, it has been suggested that the word “stressful” should be reserved for uncontrollable and/or unpredictable stimuli [53].

In the current study, we sought to examine lateralised visual inspection in horses confronted with an emotionally arousing stimulus. We examined whether horses inspected a novel, uncontrollable, and unpredictable stimulus appearing in a familiar environment preferentially with one eye over the other. Since the left eye/right hemisphere may be specialized for responding to novel or threatening stimuli, and for expressing intense emotions, we expected the horses would display a left eye (and hence right hemisphere) bias for inspecting the stimulus [30].

## Materials & methods

### 1. Animals

Ninety-eight Italian saddle horses were exposed to a novel visual stimulus in a familiar stall [54, 55]. The horses came from seven different stables in the Piemonte Region if Italy. Based on the quality of the video recordings, 77 horses (30 geldings and 47 intact females, aged 4 to 24 years) from the total sample were selected for the present study. The horses were stabled in individual stalls (ranging in size from 9 to 12 m^2^) and had paddock turnout at least three times per week. They were fed hay twice per day, concentrates three to four times per day. Water was available *ad libitum*. The horses were not provided with toys (plastic bottles, balls, etc.) in the stall [54].

### 2. Stimulus presentation

In animals, an acute emotional response and avoidance reaction can be induced by the sudden appearance of an unfamiliar stimulus [56]. In the current study, a yellow balloon (13 cm when uninflated and about 25 cm Ø when inflated, see S1 Fig) was remotely inflated near the horse inside of a familiar stall to introduce a mild, acute, unpredictable, and uncontrollable stimulus. The stimulus presentation apparatus consisted of a metal panel (40×40 cm) with a hole (3 cm Ø) in the centre, concealed by two small flaps. One end of a compressed air hose (10 m long) was inserted into this hole and an inflatable rubber balloon was attached to it, while the other end was connected to a canister of compressed air. The apparatus allowed us to rapidly inflate the balloon which the horses were not accustomed to and could not predict or control.

A webcam (QuickCam Pro 9000, Logitech, Lausanne, Switzerland) was positioned inside a second opening in the balloon presentation apparatus, 15 cm above the balloon release opening and connected to a laptop computer (K42J, ASUS, Beitou District, Taipei, Taiwan) positioned 10m away, out of sight of the focal animal. The webcam was directly aligned along the vertical axis of the inflated balloon. Therefore, the horse was recorded from viewpoint of the balloon avoiding any perspective errors that could be introduced by filming from an off-centre vantage point. We analysed the video recordings using Kinovea 0.8.15 (https://www.kinovea.org/).

Each horse was acclimated to the presence of the apparatus in the stall for 5 minutes before the onset of the trial. The experimenter then opened the compressed air valve from 10m away causing the balloon to inflate and suddenly appear. Each balloon was inflated with 2 bar of air pressure over 6s of inflation and remained inflated for 5 minutes. We conducted trials in the morning between 09:00h and 12:00h, at least 1 h after feeding (for further details see references [54] and [55]). The horses were unrestrained and free to move around the stable during the testing period. No persons were in view of the horses for the duration of the trial (10 minutes: 5 minute acclimatization plus 5 minute test).

These videos have previously been used to analyse how age affects the reactivity toward an uncontrollable and/or unpredictable stimulus [54] and to examine displacement behaviours [55] in horses. Baragli et al [54] found an age effect on the reactivity of horses towards the stimulus, with older horses showing less behavioural reactivity. Scopa et al. [55] found that behaviours linked with arousal decreased over the 5 minutes of observation suggesting habituation to the stimulus. Neither of these previous studies considered lateralisation of behaviour or sensory perception.

### 3. Behavioural measures

**Fig 1:**
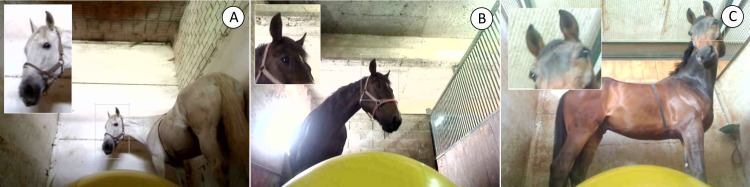
Example images of horses investigating the stimulus (an inflated balloon) with (A) their left eye, (B) their right eye, or (C) both eyes simultaneously, from the perspective of the stimulus.

We measured the amount of time each horse spent looking at the balloon exclusively with one eye (monocular investigation, the time the left or right eye was visible from the balloon’s perspective, while the contralateral eye was not) over the 5 minute trial. We considered a horse to be engaged in monocular investigation towards the stimulus (actively looking) when the auricle was directed toward the balloon (Fig 1), which was positioned in the visual hemifield [57, 58]. We also recorded eye use separately for each minute long interval during the trial to allow us to investigate whether eye use preferences changed over time.

For each horse, we calculated a laterality index (*LI* = [*right*—*left*]/[*right* + *left*]) for the duration of time spent investigating the balloon with each eye. Horses that spent more than 75% of their time in monocular inspection observing the balloon one eye over the other (LI > 0.5 = right eye preferred; LI < -0.5 = left eye preferred) over the course of the 5 minute trial were considered to show an eye use preference [59].

Horses were free to move in their stall prior to and during the trial. Consequently, the stimulus could initially appear on either side of the horse, depending which direction they were facing when the balloon was inflated. We classified the starting position of each horse based on which side of the animal was directed towards the balloon apparatus at the onset of inflation.

### 4. Data analysis

After classifying the horses as right or left lateralised based on the laterality index, we compared the number of left lateralised to the number of right lateralised horses using a binomial test. For each horse, we examined the tendency to use each eye to inspect the balloon using a linear mixed model. Eye use was used as a within subjects repeated measure (duration of monocular inspection on the right, duration of monocular inspection on the left). We included the 2-way interactions between sex, age, and the starting position of the horse at the beginning of the trial with eye use as fixed factors in the model to examine whether these factors affected the tendency to use the left or right eye. We included subject identity and home stable as random factors. The duration of inspection with each eye was Log10 transformed prior to analysis to account for the positive skew of the data. We inspected the model residuals for departures from normality using a Q-Q plot. To examine the effect of the trial time on eye use preferences, we use a linear mixed model including horse as a random factor and the minute of observation (1–5) as a repeated measure. We examined both the directional preference (laterality index = [*right*—*left*]/[*right* + *left*]) and the absolute strength of that preference (the absolute value of the laterality index) across time. We carried out the analysis using SPSS 26 (IBM, USA) for Macintosh (MacOS version 10.15.7).

### 5. Ethical note

This study was carried out in accordance with the EU Directive 2010/63/EU for animal experiments (adopted by the Italian Animal Care Act, decree Law 26/2014). The Ethical Committee on Animal Experimentation of the University of Pisa approved the experimental design (Prot. N. 0033937/2018). Consent to participation in the test was signed by the owner of each horse.

## Results

**Fig 2:**
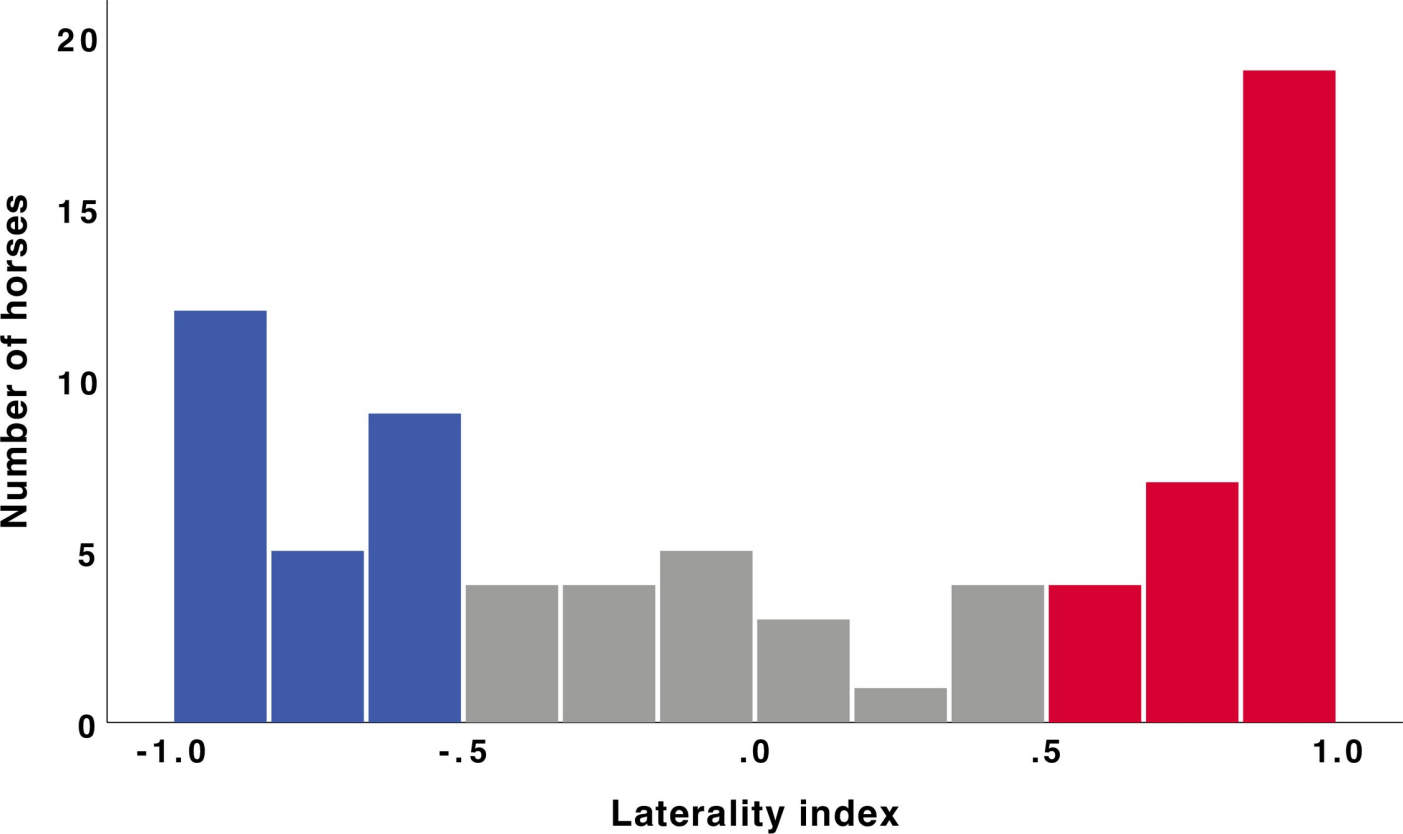
The laterality index of each horse while inspecting the balloon stimulus with one eye. A laterality index of > 0.5 indicates a right eye preference (shaded in red) while laterality index < -0.5 indicates a left eye preference (shaded in blue). Horses that showed a weak or absent preference for one eye over the other are shaded in grey. The number of horses showing left and right eye preferences did not differ (p = 0.69).

Horses spent a mean (± SEM) of 48.27 ± 4.38s with a single eye visible and 9.36±1.56s with both of their eyes visible from the perspective of the balloon. While inspecting the balloon with one eye, 56 out of 77 horses preferred to use one eye over the other (Fig 2). Of those 56 horses, 30 showed a preference for the right eye and 26 for the left eye, which did not significantly differ from chance (two-tailed binomial p = 0.69).

**Fig 3:**
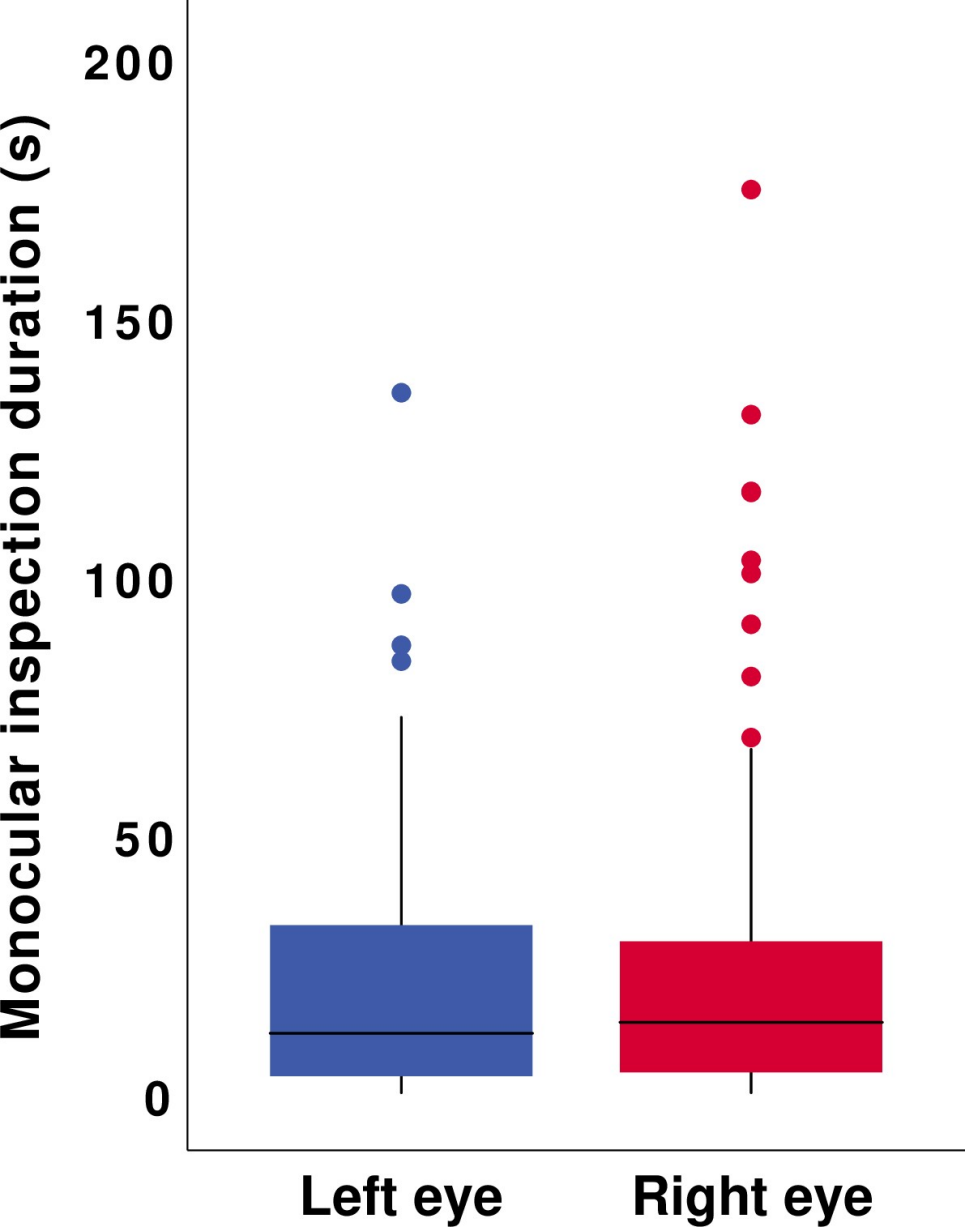
Time spent inspecting the inflated balloon stimulus using the left or right eye. Eye (left versus right) did not significantly predict the time spent in monocular inspection across horses (p = 0.47).

Across the horses, there was no significant effect of eye (right versus left) on the amount of time spent examining the balloon with one eye (F_1,148.66_ = 0.53, p = 0.47; Fig 3). Age (F_1,73_ = 0.26, p = 0.77), sex (F_1,73_ = 0.24, p = 0.79), and the starting position of the horse (F_1,73_ = 0.43, p = 0.65) did not significantly interact with eye preference, and therefore did not predict the tendency to use the right versus the left eye to inspect the balloon.

**Fig 4:**
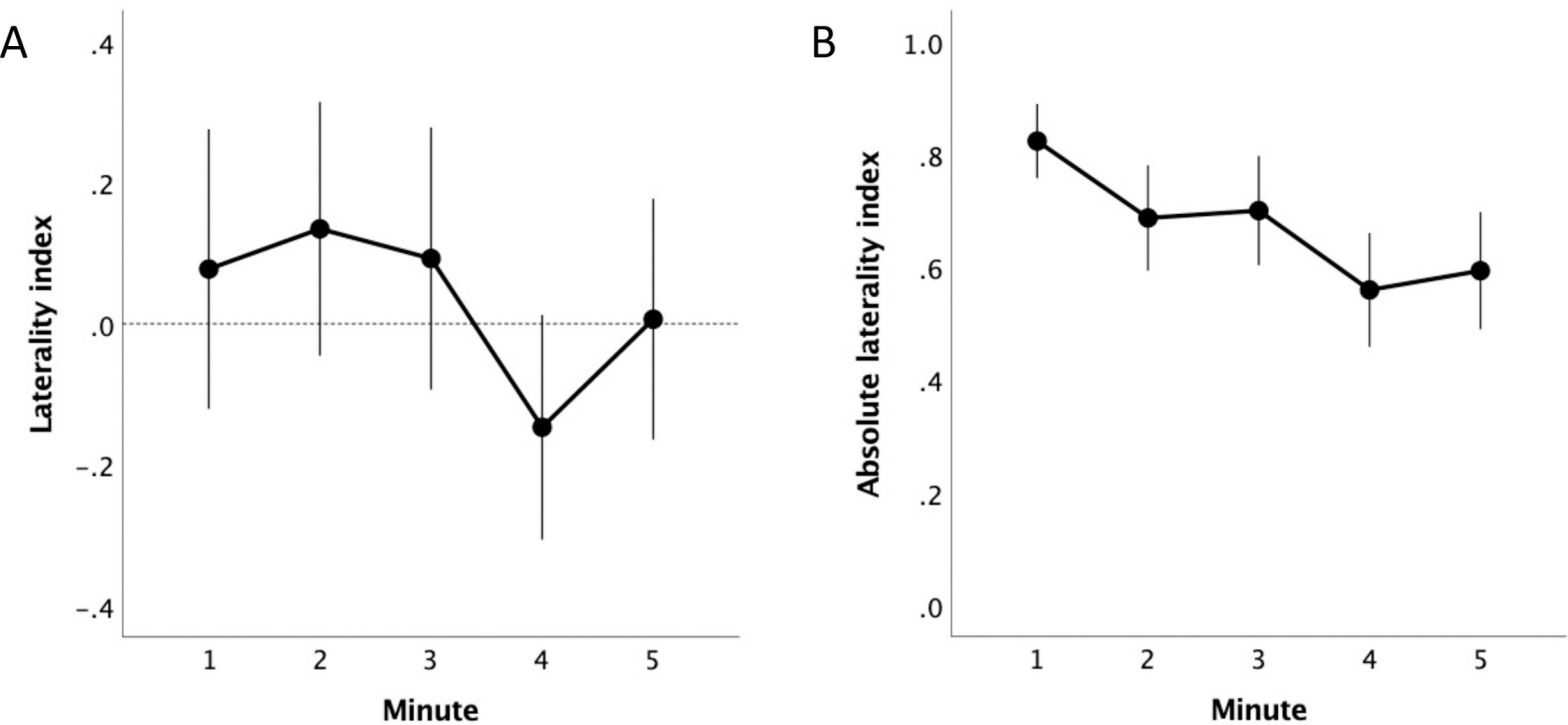
Direction and strength of eye use preference over time. (A) preference for inspecting the balloon with the left versus the right eye (laterality index). The eye use preference changed significantly across the trial (effect of minute; p = 0.01). (B) the strength of eye use preference (absolute laterality index). Horses inspected the balloon with their non-preferred eye significantly more as the trial progressed (effect of minute; p < 0.001).

There was a significant effect of time on eye use preference (F_4,90.78_ = 3.34, p = 0.012; Fig 4A) suggesting the horses’ response to the balloon changed across the trial, although there was no consistent pattern across individuals. When looking at the absolute magnitude of the preference for one eye over the other, irrespective of direction, horses inspected the balloon significantly more with their non-preferred eye as the trial progressed (F_4,134.20_ = 6.86, p < 0.001; Fig 4B).

## Discussion

We found that horses spent most of their time inspecting an unexpected novel stimulus (an inflated balloon) with one eye at a time rather using their binocular visual field. Most horses showed a clear preference for one eye over the other, however, contrary to prediction, the left eye (projecting predominantly to the right hemisphere) was not more likely to be used, and similar number of horses showed a preference for the left and right eyes. These preferences changed over the course of the trial, with the preference for one eye over the other being the strongest in the first minute of inspection, with a gradual increase in the use of the non-preferred eye over the course of the 5-minutes of observation.

Lateralisation of neural function appears to be a universal feature of nervous systems [60, 61], however the tendency for the direction of lateralised function to be aligned at the population level is highly variable between species and even populations [62–65]. The benefits of lateralised processing in the brain do not depend on population level consistency in the direction of asymmetry, and in fact consistent alignment at the population level may have costs in terms of behavioural predictability or exploitable sensory biases [66]. Social species may still be under selection for population level lateralisation if that consistency increases coordination between social fellows [67], which in turn may improve socially dependent antipredator strategies such as herding or schooling [62]. Theory predicts that social living prey species ought to show population level alignment for the detection of threats, while less social species tend to show individual level lateralisation only [68, 69]. Some previous data on horses supports this prediction [33, 35, 45], however, our results suggest that this may depend on the context, methodology, or even the specific population under study [see also: 31, 32, 34, 36, 43, 47, 70].

Stress response tests have been widely used to evaluate emotional responses in animals, since the expression of negative emotions (when a stimulus induces a negative and unpleasant state) [71] is usually well-defined and hence easier to observe than positive emotional expression [72]. Stress response tests have been widely employed in the study of functional lateralisation [31, 44, 73], however, these tests have yielded conflicting results [74]. This lack of consistency is a general phenomenon in the investigation of emotional states and is not unique to studies of sensory laterality [72]. Emotion is a subjective experience, and therefore, individual variation in the reaction to a particular stimulus is to be expected [75]. The subjective perception of a stimulus will determine the degree to which it acts as a stressor [76] and how it is approached by the animal. Individual variation in the evoked motivation or emotional response towards a given stimulus could explain variation in the lateralisation of visual investigation of that stimulus [77, 78]. For example, if some horses found the balloon frightening, while others thought interesting in a more neutral or even positive way, then we may expect to see individual variation in the direction and strength of laterality among animals, fitting with the patterns we observed, even in the presence of a strong underlying population level lateralisation of for investigating negatively valent stimuli. The horses in our study spent approximately 20% of the trial visually inspecting the balloon on average, although this was highly variable between individuals. This relatively brief period of visual inspection could suggest that at least some individuals did not find the balloon to be as emotionally arousing as we had expected. Just over half of our sample showed avoidance behaviours, moving rapidly away from the stressor [54]. In the concept of stress proposed by Koolhaas et al [53], a stimulus can be defined as a “stressor” when it is uncontrollable and unexpected, thus causing arousal, which remains even when the stimulus is removed, suggesting that the animal may have perceived the stimulus as a threat. In our study, the inflated balloon meets these *a priori* requirements (unpredictability and uncontrollability) of a stressor. However, we did not have an independent measure of stress response in tested horses (e.g., a physiological measure) and therefore, it is possible that the balloon did not induce stress or was perceived as stressful by some but not all horses.

Our current results are not necessarily at odds with previous studies that have found population level consistency in the direction of laterality among horses. Our sample size was large and heterogeneous, and despite rigorous control of experimental procedures, the differing backgrounds of the animals included in our study may have led to greater variation in the direction of lateralisation exhibited. Studies carried out with a smaller number of subjects drawn from a homogenous population maybe be more likely to show lateralization at the population level due to a more uniform response to the stimulus. Consistent handling, management, and/or training history, could increase consistency in sensory laterality at the population level.

Horses are conventionally trained and handled from their left side including leading, saddling, percutaneous injections, and other common procedures [35, 79]. This prior experience could lead horses to react differently when a stimulus appears on their left side compared to their right side. This domestication bias has been suggested as a possible explanation for some population level asymmetries in horses [31]. Recently however, it has been shown that initial training does not affect sensory lateralisation [79]. The lack of population level consistency that we observed supports this contention. Furthermore, it has been suggested that sensory and motor laterality may be strengthened with age because of training [80], while other authors suggest that training may reduce the natural emotional asymmetry to human approach shown by horses [35]. The horses tested in our study were all trained from left side, per conventional stable management practice, and we did not detect any effect of age across a range of 4–24 years, suggesting that training history did not have a major effect in our sample.

We tested each horse only once, raising the question of how repeatable the eye use biases we observed would be within animals across time [81–83]. We found significant changes in the pattern of eye use across the 5-minutes of observation; notably, the horses used their non-preferred eye to inspect the stimulus more often as the trial progressed. Whether this trend would continue as the balloon became increasingly more familiar is an open question, but we predict that eventually the horse would largely cease to attend to the balloon as it became clear that it was not a threat, and any further visual inspection would not be biased to one eye over the other. Due to the importance of emotional state on lateralised processing, and the effects of habituation, a direct retest using the same setup is unlikely to be especially informative. Future work should aim to test eye use preferences across multiple contexts using different emotionally valent stimuli to determine the within individual consistency of these characteristics across contexts and over time.

We tested the horses in their home stall which is between 9 and 12 square meters square. Although offering plenty of space to move around, this confinement may have affected our results by limiting the ability for horses to escape from the stimulus, something that 57% of the animals nevertheless attempted to do [54]. To our knowledge only one paper has examined the influence of space constraints on laterality mammals, Zucca et al. [84] found that space availability affected variation in the laterality strength and direction in donkeys. When coping with a potential threat in a restricted space, animals should quickly evaluate the stimulus to determine the potential danger, regardless of the side of inspection, which may suppress population level lateralised tendencies.

## Conclusions

In the current study, we found that individual horses responded to a novel and unexpected stimulus by examining it primarily with one eye. Across horses, the preferred eye (right or left) was not consistent, nor did sex or age affect the preferred eye. This individual variation suggests that training history is not the predominant causal factor in determining visual laterality in the horse. Horses showed a decreasing preference for using one eye over the other to inspect the stimulus as the trial progressed, suggesting that the horses may have habituated to the stimulus. Our study suggests that in at least in some testing contexts, horses appear to show individual but not population level laterality. We highlight the necessity for carefully designed observation methods in which data is recorded from a vantage point in line with the stimulus and the midline of the animal to avoid perspective errors in eye use data collection. We also suggest that stimuli assumed to act as stressors may not always do so for all sampled individuals, and ideally should be verified with physiological data in future work.

## Supporting information

S1 Fig The balloon stimulus used in the study: (A) uninflated, (B) inflated and viewed from the lateral perspective, (C) inflated a viewed from a frontal perspective.
(DOCX)

S1 File (XLSX)
