# Supplementary material for: Horses show individual level lateralisation when inspecting an unfamiliar and unexpected stimulus
Source: PLoS One. 2021 Aug 5;16(8):e0255688. doi: 10.1371/journal.pone.0255688 (PMC8341651; doi:10.1371/journal.pone.0255688)
Supplement: S1 Fig — The balloon stimulus used in the study: (A) uninflated, (B) inflated and viewed from the lateral perspective, (C) inflated a viewed from a frontal perspective. (DOCX) [file pone.0255688.s001.docx]

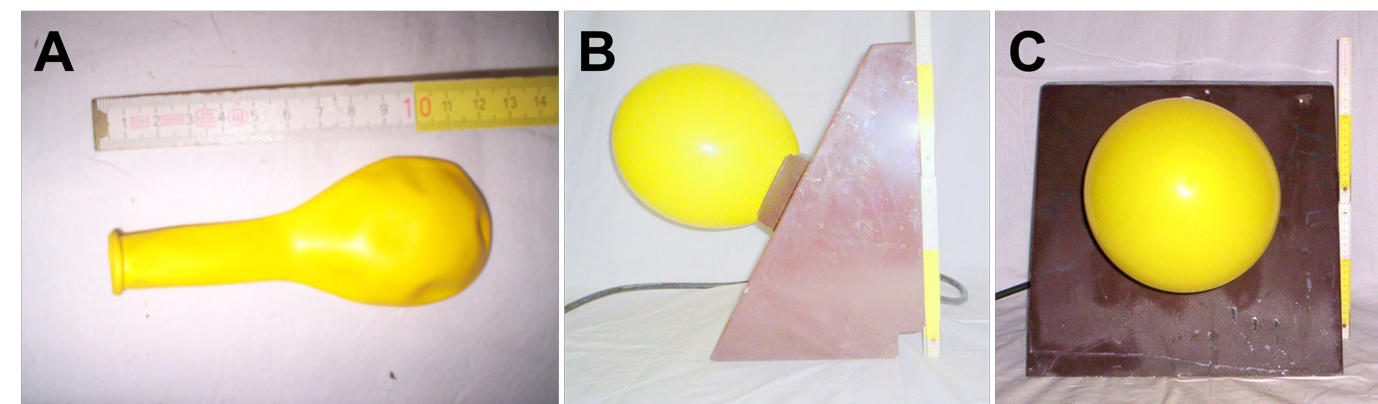


**S1 Fig.** The balloon stimulus used in the study: (A) uninflated, (B) inflated and viewed from the lateral perspective, (C) inflated a viewed from a frontal perspective.
